# Samuel Hobart Dorr, Ph. G., Ph. M.

**Published:** 1912-04

**Authors:** 


### OBITUARY

---

Samuel Hobart Dorr, Ph. G., Ph. M., a graduate of the Department of Pharmacy of the University of Buffalo, and for many years Professor of Microscopy, died March 18, 1912, after a lingering illness, terminating in pneumonia. He was born in Scottsville, July 8, 1850.

Mr. Dorr was known to many of the medical profession as a thoroughly competent pharmacist, to all who met him, as a genial though dignified gentleman. His modesty was such that only those associated with him as colleague or student, or those of similar tastes, knew of his researches in pharmacognosy and botany.

